# The Impact of High-Fat Diet on Mitochondrial Function, Free Radical Production, and Nitrosative Stress in the Salivary Glands of Wistar Rats

**DOI:** 10.1155/2019/2606120

**Published:** 2019-07-04

**Authors:** Anna Zalewska, Dominika Ziembicka, Małgorzata Żendzian-Piotrowska, Mateusz Maciejczyk

**Affiliations:** ^1^Department of Conservative Dentistry, Medical University of Bialystok, Poland; ^2^Department of Public Health, Medical University of Bialystok, Poland; ^3^Department of Hygiene, Epidemiology and Ergonomics, Medical University of Bialystok, Poland; ^4^Department of Physiology, Medical University of Bialystok, Poland

## Abstract

Oxidative stress plays a crucial role in the salivary gland dysfunction in insulin resistance; however, the cause of increased free radical formation in these conditions is still unknown. Therefore, the aim of the study was to investigate the effect of high-fat diet (HFD) on the mitochondrial respiratory system, prooxidant enzymes, ROS production, and nitrosative/oxidative stress in the submandibular and parotid glands of rats. The experiment was performed on male Wistar rats divided into two groups (*n* = 10): control and HFD. The 8-week feeding of HFD affects glucose metabolism observed as significant increase in plasma glucose and insulin as well as HOMA-IR as compared to the control rats. The activity of mitochondrial Complex I and Complex II+III was significantly decreased in the parotid and submandibular glands of HFD rats. Mitochondrial cytochrome c oxidase (COX) activity and the hydrogen peroxide level were significantly increased in the parotid and submandibular glands of the HFD group as compared to those of the controls. HFD rats also showed significantly lower reduced glutathione (GSH) and reduced : oxidized glutathione (GSH : GSSG) ratio, as well as a higher GSSG level in the parotid glands of HFD rats. The activity of NADPH oxidase, xanthine oxidase, and levels of oxidative/nitrosative stress (malonaldehyde, nitric oxide, nitrotyrosine, and peroxynitrite) and inflammation/apoptosis (interleukin-1*β* and caspase-3) biomarkers were statistically elevated in the HFD group in comparison to the controls. HFD impairs mitochondrial function in both types of salivary glands by enhancing ROS production, as well as stimulating inflammation and apoptosis. However, free radical production, protein nitration, and lipid peroxidation were more pronounced in the parotid glands of HFD rats.

## 1. Introduction

One of the most important problems of modern diabetology is a growing incidence of type 2 diabetes. Two basic pathological defects are observed in the pathogenesis of this disease, namely, reduced insulin secretion and reduced sensitivity to this hormone in its target tissues, i.e., insulin resistance (IR). Insulin resistance is also an important element of the so-called metabolic syndrome, so it is considered an extremely serious health problem.

The occurrence of insulin resistance is explained by the existence of insulin antibodies, accelerated insulin degradation, and/or insulin transmission disorders. In the pathogenesis of this phenomenon as well as its complications, the role of oxidative stress (OS) is also emphasized [1]. By definition, oxidative stress is a situation in which the temporarily or chronically elevated level of reactive oxygen species (ROS) cannot be neutralized by antioxidant systems, resulting in disturbances of cell metabolism and degradation of cellular components [2].

In our earlier studies [3], we demonstrated that insulin resistance induced by high-fat diet disturbs the antioxidant systems, primarily of the parotid glands and, to a lesser extent, the submandibular glands. The weakening of the antioxidant defense of salivary glands in the course of IR was most probably a result of their wear in the process of ROS elimination, as well as an effect of oxidative modifications of polypeptide chains of antioxidant enzymes. Kołodziej et al. [4] showed that insulin resistance causes oxidative modifications of biomolecules in the salivary glands, including proteins, lipids, and DNA. Moreover, oxidative damage to the salivary glands has been proven to be a part of mechanisms leading to dysfunction of the salivary glands of rats in the course of insulin resistance [4]. The authors also demonstrated that disturbed salivary redox balance was not the result of systemic redox balance disturbances. However, the sources of ROS in the salivary glands in the course of IR are still unknown.

Saliva produced in the salivary glands plays a crucial role in maintaining good health, not only in the oral cavity. Saliva ensures homeostasis and moisturizes and cleanses mucous membranes and teeth. It participates in the initial stage of carbohydrate digestion as well as facilitates the formation and swallowing of food pieces. Buffer systems of saliva maintain constant pH, protecting teeth against decay and erosion [5, 6]. From the point of view of redox balance, saliva contains very effective antioxidant systems that constitute the first line of defense of the gastrointestinal tract against ROS [7].

Bearing in mind the proven role of oxidative stress in salivary gland dysfunction in the course of IR induced by a high-fat diet and the role of salivary glands in maintaining redox homeostasis, it seems important to learn about the mechanisms leading to disrupting this balance. It is highly probable that, as in the case of the skeletal muscles of rats [8], one of the causes of OS in the salivary glands is increased mitochondrial and cytoplasmic ROS production, which exceeds the antioxidant capacity of these glands. However, so far, there have been no studies investigating the impact of high-fat-diet-induced insulin resistance on mitochondrial function in the salivary glands of rats. There are also no available studies analyzing the changes in the activity of other enzymatic intracellular sources of ROS and nitrosative stress in these glands. Therefore, the aim of our study was to investigate the effect of IR induced with high-fat diet on the mitochondrial respiratory system, the activity of prooxidant enzymes, ROS production, and nitrosative stress in salivary glands of insulin-resistant rats.

## 2. Materials and Methods

The protocol of the study was approved by the Local Ethical Committee for Animal Experiments of the Medical University of Olsztyn, Poland (protocol number 21/2017).

### 2.1. Animals

The experiment was performed on male Wistar rats (cmdb outbred rats, 5 weeks of initial age) obtained from the Center of Experimental Medicine, Medical University of Bialystok, Poland. Constant environmental conditions, i.e., 12-hour light/dark cycle, stable temperature of 20 ± 2°C, and relative humidity of 65–70%, were maintained throughout the entire study. After one week of an adaptation period, the rats were randomly divided into two equally numbered groups of 10 animals (*n* = 10):

1. Control (C) group
2. High-fat diet (HFD) group

For 8 consecutive weeks, rats from the C group were fed (*ad libitum*) a regular diet containing 10.3% fat, 24.2% protein, and 65.5% carbohydrates (kcal) (Agropol, Motycz, Poland), while rats from the HFD group had a high-fat diet containing 59.8% fat, 20.1% protein, and 20.1% carbohydrates (kcal) (Research Diets Inc., New Brunswick, NJ, USA, D12492) [1]. Food consumption and body weight were monitored every 3 days.

After 8 weeks of the experiment, all the rats were fasted for 12 hours and anesthetized with a sodium phenobarbital injection (80 mg/kg body weight, intraperitoneally). The body mass index (BMI) was measured based on the weight and length from the tip of the nose to the anus [9, 10]. The experiment also included the analysis of fasting tail-blood glucose (Accu-Chek Roche, Bayer, Germany) as well as the measurement of nonstimulated (NWS) and stimulated (SWS) salivary flow, as described earlier [11]. NWS was measured for 15 minutes using preweighed cotton balls inserted into the oral cavity underneath the tongue [11]. To evaluate SWS, rats had pilocarpine hydrochloride injected (5 mg/kg body weight, intraperitoneally) and their salivary secretion was examined for 5 minutes. NWS and SWS were determined based on the difference in the initial and final weight of the cotton balls. It was assumed that 1 mg of whole saliva was equal to 1 *μ*L [12] in volume. Then, blood samples were collected from the abdominal aorta, and salivary glands (both parotid and submandibular) were taken by an experienced laboratory technician. The tissues were freeze-clamped with aluminum tongs, precooled in liquid nitrogen, and stored at -80°C until the redox assays (but not longer than 2 months) [13, 14].

The level of plasma insulin was measured using a commercial ELISA kit (Shibayagi Co., Gunma, Japan) according to the manufacturer's instructions. Insulin sensitivity was evaluated by the HOMA-IR index (homeostasis model assessment of insulin resistance = (fasting insulin (U/mL) × fasting glucose (mM))/22.5) [10]. Plasma free fatty acid (FFA) levels were analyzed by gas chromatography (GC) [15].

### 2.2. Salivary Gland Preparation

On the day of biochemical assays, the tissue samples were slowly thawed at 4°C, fragmented, weighed, and divided into two equal parts. One of them was diluted in ice-cold phosphate-buffered saline (PBS, 0.02 M, pH 7.4) at a ratio of 1 : 10 (*w*/*v*). The salivary glands were homogenized on ice with a glass tissue homogenizer (Omni TH, Omni International, Kennesaw, GA, USA), sonicated (1800 J/sample, 20 s *×* 3; UP 400S, Hielscher, Teltow, Germany), and then centrifuged (12,000 × g, 20 min, 4°C; MPW Med Instruments, Warsaw, Poland) to collect the supernatant and be immediately assayed [3]. In order to prevent sample oxidation and proteolysis, butylated hydroxytoluene (BHT; 10 *μ*L 0.5 M BHT in acetonitrile/1 mL PBS) and proteolysis inhibitors (Complete Mini Roche, France) were added [4]. The other part of the salivary gland samples was diluted in mitochondria isolation buffer.

### 2.3. Mitochondria Isolation

Salivary glands were homogenized (1 : 10, *w*/*v*) with a Teflon-on-glass electric homogenizer in an ice-cold mitochondria isolation buffer containing 250 mM sucrose, 5 mM Tris-HCl, and 2 mM EGTA (ethylene glycol bis(2-aminoethyl)tetraacetic acid) (pH 7.4) [16, 17]. To prevent protein degradation and proteolysis, protease inhibitors were added [18]. The homogenate was centrifuged (500 × g, 10 min, 4°C) to remove the nuclei and cell debris, and the resulting supernatant was centrifuged twice at 8000 × g for 10 minutes at 4°C. The mitochondria pellet was resuspended in an isolation buffer and immediately processed [19].

### 2.4. ROS Production

The activity of NADPH oxidase (NOX, E.C. 1.6.3.1) was measured by a luminescence assay using lucigenin as an electron acceptor [20]. One unit of NOX activity was defined as the quantity of enzyme required to release 1 nmol of the superoxide radical for 1 minute.

The activity of xanthine oxidase (XO, E.C. 1.17.3.2.) was analyzed based on uric acid (UA) formation, measuring the increase in its absorbance at a 290 nm wavelength [21]. One unit of XO activity was defined as the amount of enzyme required to release 1 *μ*mol of UA for 1 minute.

ROS production was estimated spectrofluorimetrically using 2,7-dichlorodihydrofluorescein diacetate (DCFH-DA), which is deesterified to 2,7-dichlorodihydrofluorescein (DCFH) by oxygen free radicals [22]. The ROS formation rate was calculated from the calibration curve for DCFH.

NOX, XO, and DCFH levels were measured immediately after sample collection [23].

### 2.5. Mitochondrial Activity

The activity of Complex I (E.C. 1.6.5.3) was assayed colorimetrically based on 2,6-dichloroindophenol (DCIP) reduction by electrons accepted from decylubiquinol (coenzyme Q_1_), reduced after oxidation of NADH (reduced form of nicotinamide adenine dinucleotide) by Complex I [16].

The activities of Complex II (E.C. 1.3.5.1) and Complex II+III (E.C. 1.10.2.2) were analyzed according to the method by Rustin et al. [24]. For this purpose, the activities of succinate-ubiquinone reductase and succinate-cytochrome c reductase were measured colorimetrically.

The activity of cytochrome c oxidase (COX) was estimated colorimetrically by measuring the oxidation of reduced cytochrome c at a 550 nm wavelength [25].

The activity of citrate synthase (CS) was analyzed colorimetrically in the reaction with 5-thio-2-nitrobenzoic acid which is generated from 5,5′-dithiobis-(2-nitrobenzoic acid) during CS biosynthesis [26].

The production of mitochondrial hydrogen peroxide (H_2_O_2_) was assayed using the Amplex™ red-horseradish peroxidase method [27]. Horseradish peroxidase (HRP, 2 U/mL) catalyzes the H_2_O_2_-dependent oxidation of nonfluorescent Amplex red (80 *μ*mol/L) to fluorescent resorufin red. 9 mM succinate and 5 mM glutamate + malate were used as a substrate. The rate of H_2_O_2_ production was calculated based on a standard curve of H_2_O_2_ stabilized solution. Fluorescence was measured at an excitation wavelength of 545 nm and an emission wavelength of 590 nm [17].

The ADP/ATP ratio was measured using a commercial bioluminescent kit (ADP/ATP Ratio Assay Kit ab65313; Abcam, USA), according to the manufacturer's instructions. In this assay, luciferase catalyzes the conversion of ATP and luciferin to light, which is measured using a luminometer. The ADP level is estimated by its conversion to ATP that is subsequently detected using the same reaction.

The activities of Complexes I, II, and II+III, COX, CS, ADP/ATP ratio, and H_2_O_2_ production rate were assayed in isolated mitochondria, immediately after sample preparation.

### 2.6. Glutathione Metabolism

The level of total glutathione was assayed using the enzymatic reaction with NADPH (reduced form of nicotinamide adenine dinucleotide phosphate), DTNB (5,5′-dithiobis-(2-nitrobenzoic acid)), and glutathione reductase (data not shown) [28].

The level of oxidized glutathione (GSSG) was estimated similar to the assay performed for the total glutathione, the difference being that prior to the determination, the samples had been thawed and neutralized to pH 6–7 with 1 M chlorhydrol triethanolamine and then incubated with 2-vinylpyridine [28]. The level of reduced glutathione (GSH) was calculated from the difference between the level of total glutathione and GSSG [28].

The redox (oxidation/reduction) state was calculated using the formula GSH^2^/GSSG [29].

The activity of glutathione peroxidase (GPx) was assayed colorimetrically based on the conversion of NADPH to NADP^+^ [30] The absorbance was measured at 340 nm. It was assumed that one unit of GPx activity catalyzes the oxidation of 1 mmol NADPH for 1 minute.

The activity of glutathione reductase (GR) was estimated colorimetrically by measuring the decrease in absorbance of NADPH [31]. The absorbance was measured at 340 nm. One unit of GR activity was defined as the amount of enzyme that catalyzes the oxidation of 1 *μ*mol NADPH for 1 minute.

### 2.7. Oxidative and Nitrosative Stress

The level of malonaldehyde (MDA) was measured colorimetrically using the thiobarbituric acid reactive substance (TBARS) method with 1,1′,3,3′-tetraethoxypropane as a standard [32].

The level of nitric oxide (NO) was measured colorimetrically using sulfanilamide and N-(1-naphthyl)-ethylenediamine dihydrochloride. The absorbance of the obtained product was measured at a 490 nm wavelength [33].

The level of peroxynitrite was analyzed colorimetrically based on peroxynitrite-mediated nitration resulting in the formation of nitrophenol [34]. The absorbance of the obtained complex was measured at a 320 nm wavelength.

The level of nitrotyrosine was evaluated using enzyme-linked immunosorbent assay (ELISA) (Nitrotyrosine ELISA; Immundiagnostik AG, Bensheim, Germany) according to the manufacturer's instructions.

### 2.8. Inflammation and Apoptosis

The level of interleukin-1*β* (IL-1*β*) was measured using a commercial ELISA kit (IL-1*β* ELISA kit, R&D Systems; Minneapolis, USA) in accordance with the manufacturer's instructions.

The activity of caspase-3 (CAS-3, EC 3.4.22.56) was analyzed colorimetrically using Ac-Asp-Glu-Val-Asp-p-nitroanilide as a substrate [35]. The amount of p-nitroaniline released by CAS-3 activity was measured at a 405 nm wavelength.

### 2.9. Protein Assay

The content of total protein was measured using the bicinchoninic acid (BCA) method with bovine serum albumin as a standard (Pierce BCA Protein Assay, Thermo Fisher Scientific, Rockford, IL, USA).

### 2.10. Statistical Analysis

The data was analyzed using GraphPad Prism 7 for Mac (GraphPad Software, La Jolla, USA). The statistical significance was defined as *p* ≤ 0.05. To confirm the normal distribution of the results, the Shapiro-Wilk and Kolmogorov-Smirnov tests were used. The results were expressed as mean ± standard deviations (SD). Unpaired Student's *t*-test and Pearson's correlation method were also implemented. The sample size was established based on the pilot study conducted earlier. The statistical power of the test was assumed to equal 0.9.

## 3. Results

### 3.1. General Characteristics

8 weeks of feeding a high-fat diet caused significant increase in the body weight and BMI index of the rats from the study group compared to the control rats fed standard chow (*p* = 0.0003 and *p* = 0.0012, respectively). The high-fat diet treatment affects glucose metabolism, as evidenced by the fact that we observed significant increase in blood glucose (*p* = 0.00001) and plasma insulin (*p* = 0.00001) concentrations as well as HOMA-IR (*p* = 0.0002) as compared to the control rats fed a regular diet. The content of free fatty acids was considerably higher in the serum of rats fed a high-fat diet compared to the concentration of these acids in the serum of control rats (*p* = 0.00001). Although the rats from the HFD group consumed significantly less food (*p* = 0.003), the energy value of their chow was considerably higher than that in the control group (*p* = 0.0005) (Table 1).

Despite the fact that the weight of the parotid glands was significantly higher in the HFD group vs. the control (*p* = 0.02), the rate of stimulated saliva secretion in this group was much lower compared to that in the control group (*p* = 0.0003). The weight of the submandibular glands and the rate of nonstimulated saliva secretion did not differ in both studied groups. The concentration of proteins in the parotid (*p* = 0.0005) as well as submandibular (*p* = 0.002) glands of HFD rats was significantly decreased compared to that in the respective control glands (Table 1).

### 3.2. Mitochondrial Respiratory Complex Activity

**Figure 1:**
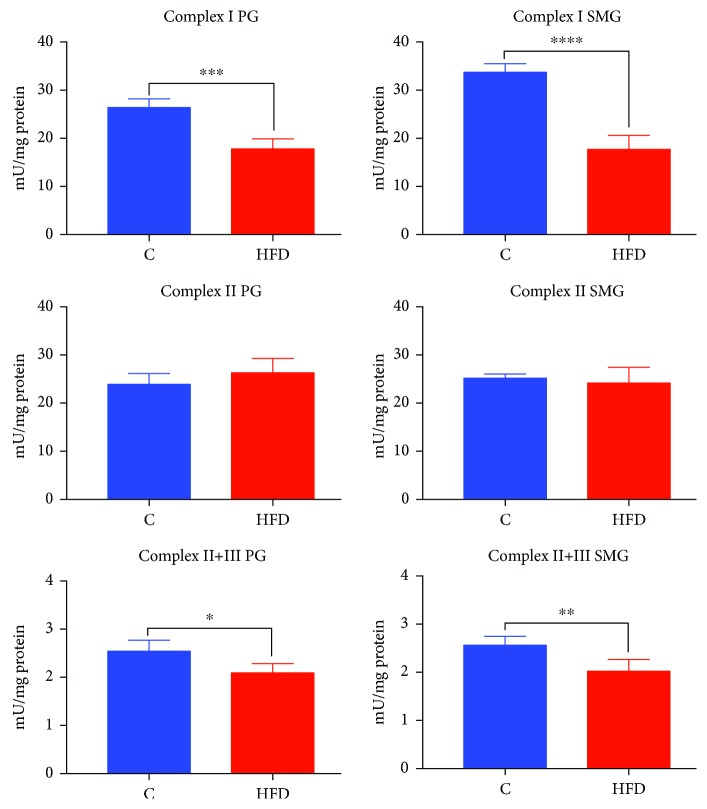
Mitochondrial respiratory complex activity in the parotid and submandibular glands of HFD (*n* = 10) and control (*n* = 10) rats. PG: parotid glands; SMG: submandibular glands; C: control group; HFD: high-fat diet group. ^∗^*p* < 0.05, ^∗∗^*p* < 0.005, ^∗∗∗^*p* < 0.0005, and ^∗∗∗∗^*p* < 0.0001. All determinations were made in duplicate.

The activity of parotid gland Complex I (↓32%, *p* = 0.0001) and Complex II+III (↓18%, *p* = 0.0130) was significantly decreased in the HFD group as compared to the controls. A similar relationship was observed in the submandibular glands of HFD rats: the activity of Complex I (↓47%, *p* < 0.0001) and Complex II+III (↓21%, *p* = 0.0060) was considerably lower in relation to that of the control rats. Both in the submandibular and parotid glands of the HFD group, Complex II activity was not different from the corresponding salivary glands of the control rats (Figure 1).

### 3.3. COX and CS Activity, ADP/ATP Ratio, and Mitochondrial H_2_O_2_ Production

**Figure 2:**
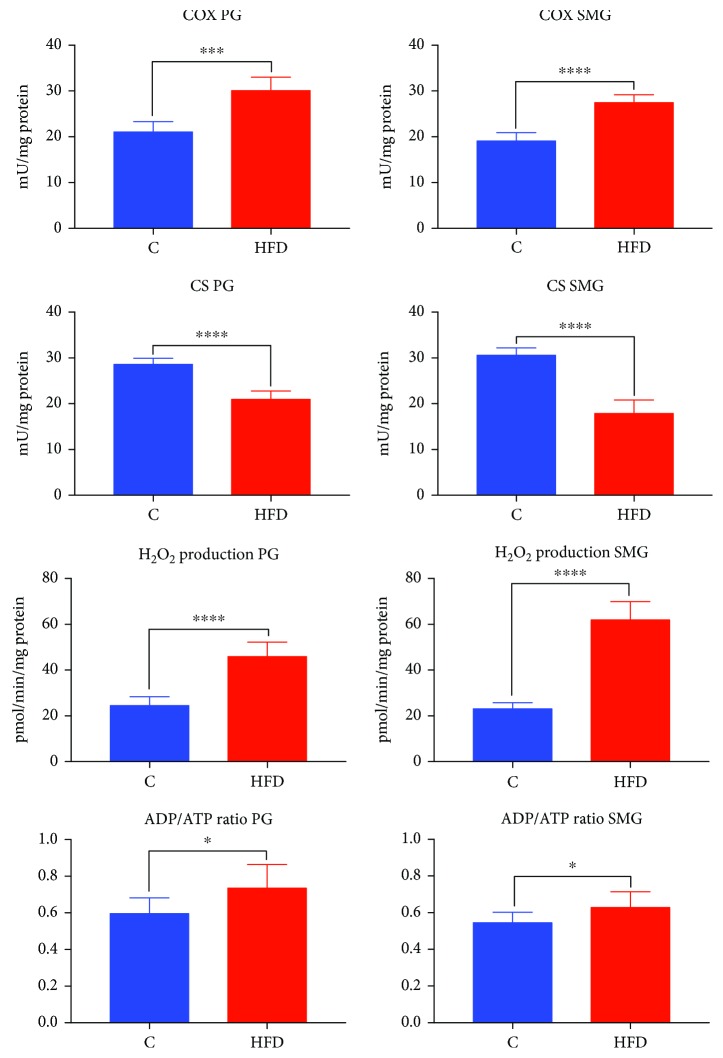
COX and CS activity, mitochondrial H_2_O_2_ production, and ADP/ATP ratio in the parotid and submandibular glands of HFD (*n* = 10) and control (*n* = 10) rats. PG: parotid glands; SMG: submandibular glands; C: control group; HFD: high-fat diet group; COX: cytochrome c oxidase; CS: citrate synthase; H_2_O_2_: hydrogen peroxide. ^∗^*p* < 0.05, ^∗∗∗^*p* < 0.0005, and ^∗∗∗∗^*p* < 0.0001. All determinations were made in duplicate.

Mitochondrial COX activity in the parotid (↑42%, *p* = 0.0003) and submandibular (↑43%, *p* < 0.0001) glands of HFD rats was significantly increased compared to that of the controls (Figure 2).

Mitochondrial CS activity in the parotid (↓27%, *p* < 0.0001) and submandibular (↓40%, *p* < 0.0001) glands of rats fed high-fat diet was significantly reduced in relation to the CS activity in the mitochondria of the controls (Figure 2).

The mitochondrial H_2_O_2_ production in the parotid (↑85%, *p* < 0.0001) and submandibular (↑163%, *p* < 0.0001) glands of HFD rats was higher compared to that of the control group (Figure 2).

ADP/ATP ratio was significantly higher in the parotid and submandibular glands of HFD rats compared to those of the control group (↑, *p* = 0.0167 and *p* = 0.0256, respectively) (Figure 2).

### 3.4. NOX and XO Activity and DCFH-DA Production in the Parotid and Submandibular Glands

**Figure 3:**
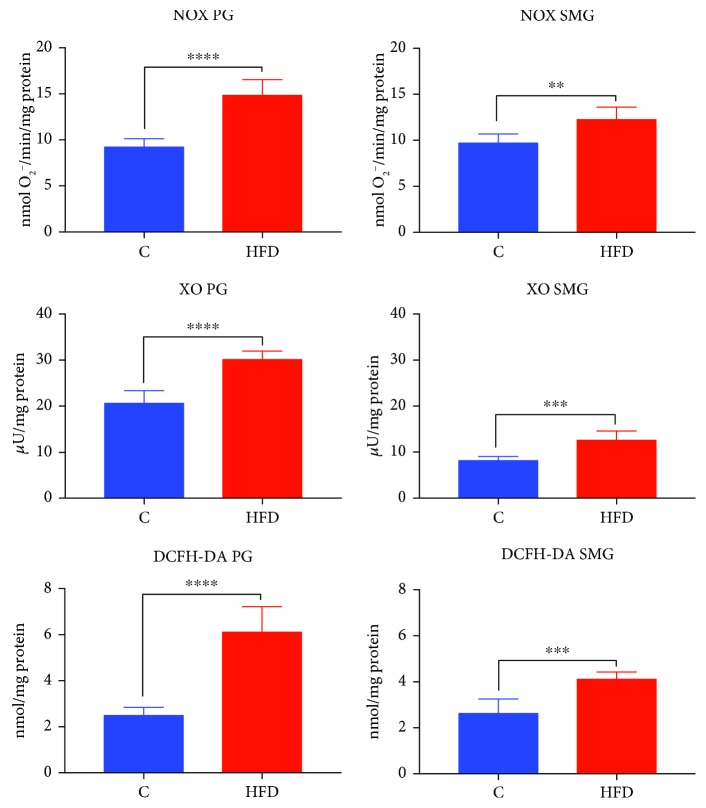
Prooxidant enzymes and free radical production in the parotid and submandibular glands of HFD (*n* = 10) and control (*n* = 10) rats. PG: parotid glands; SMG: submandibular glands; C: control group; HFD: high-fat diet group; NOX: NADPH oxidase; XO: xanthine oxidase; DCFH-DA: 2,7-dichlorodihydrofluorescein diacetate assay for free radical production. ^∗∗^*p* < 0.005, ^∗∗∗^*p* < 0.0005, and ^∗∗∗∗^*p* < 0.0001. All determinations were made in duplicate.

Determination of NOX activity in the parotid (↑59%, *p* < 0.0001) as well as submandibular (↑26%, *p* = 0.0030) glands showed higher activity of this enzyme in the HFD group compared to the control (Figure 3).

XO activity was significantly higher in the parotid (↑45%, *p* < 0.0001) and submandibular (↑52%, *p* = 0.0006) glands of HFD rats compared to those of the rats fed a regular diet (Figure 3).

DCFH-DA production was significantly more expressed in the parotid (↑142%, *p* < 0.0001) and submandibular (↑55%, *p* = 0.0002) glands of HFD rats compared to those of the control rats (Figure 3).

### 3.5. Glutathione Metabolism in the Parotid and Submandibular Glands

**Figure 4:**
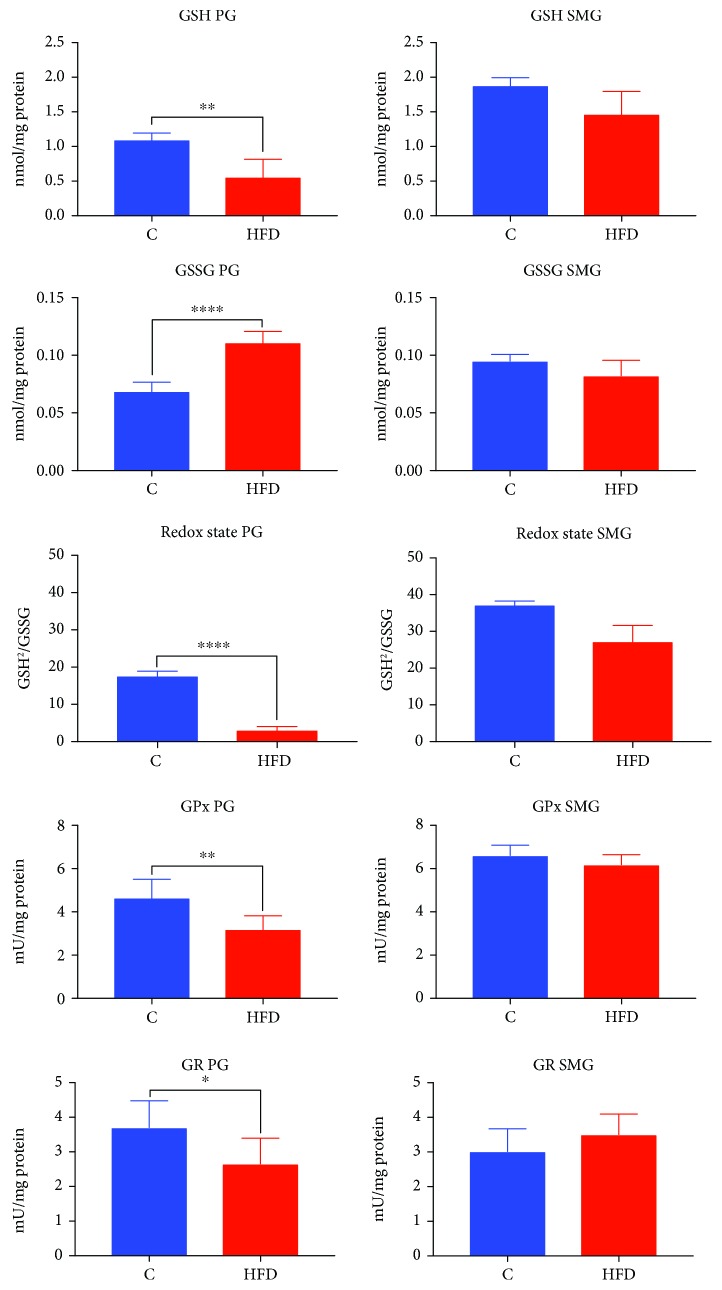
Glutathione metabolism in the parotid and submandibular glands of HFD (*n* = 10) and control (*n* = 10) rats. PG: parotid glands; SMG: submandibular glands; C: control group; HFD: high-fat diet group; GSH: reduced glutathione; GSSG: oxidized glutathione. ^∗^*p* < 0.05, ^∗∗^*p* < 0.005, ^∗∗∗^*p* < 0.0005, and ^∗∗∗∗^*p* < 0.0001. All determinations were made in duplicate.

HFD rats showed significantly reduced GSH and redox rate in the parotid glands (↓49%, *p* = 0.0022, and ↓70%, *p* ≤ 0.0001, respectively) as well as a reduced GSH level in the submandibular glands (↓22%, *p* = 0.0278) compared to the control group (Figure 4).

GSSG content was significantly increased in the parotid glands (↑57%, *p* ≤ 0.0001) of rats fed high-fat diet, while in the submandibular glands, it did not differ from the controls (Figure 4).

GPx and GR activities were significantly reduced in the parotid glands of HFD rats compared to those of the control group (↓, *p* = 0.0072, and ↓, *p* ≤ 0.0232, respectively), while in the submandibular glands, they did not differ significantly (Figure 4).

### 3.6. Inflammation and Apoptosis in the Parotid and Submandibular Glands

**Figure 5:**
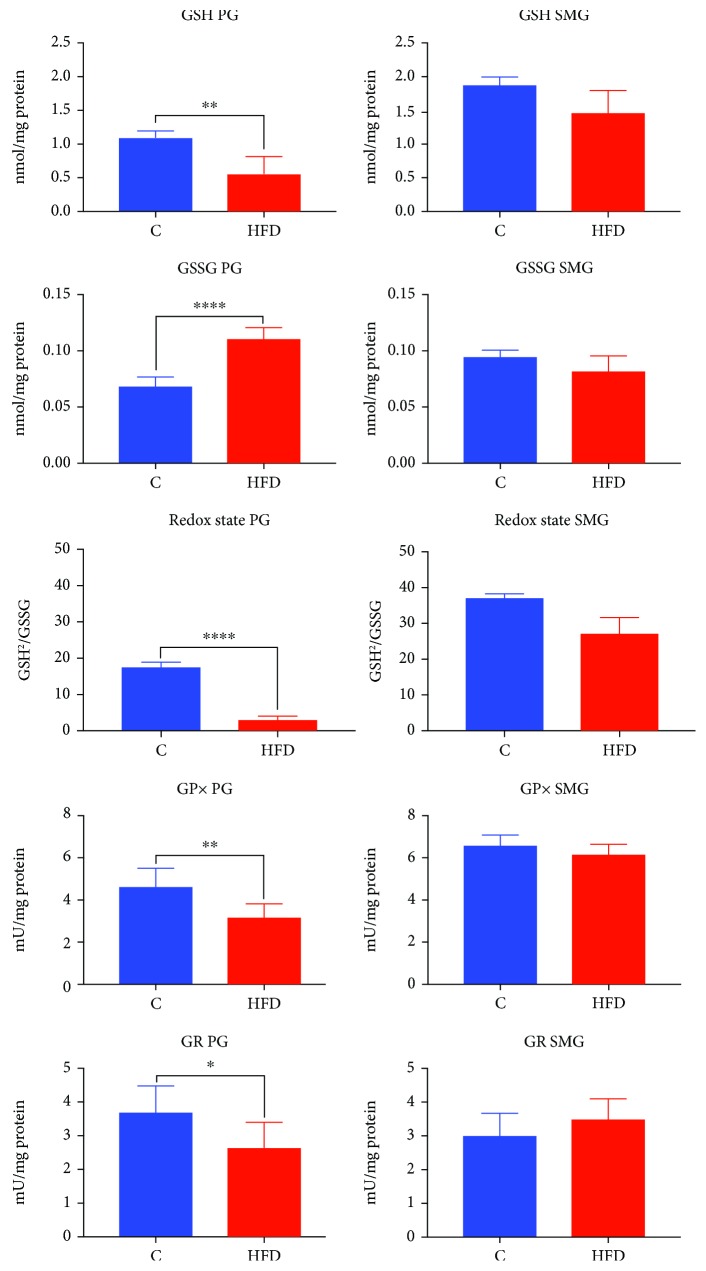
Inflammation and apoptosis biomarkers in the parotid and submandibular glands of HFD (*n* = 10) and control (*n* = 10) rats. PG: parotid glands; SMG: submandibular glands; C: control group; HFD: high-fat diet group; IL-1*β*: interleukin-1*β*; CAS-3: caspase-3. ^∗∗^*p* < 0.005, ^∗∗∗^*p* < 0.0005, and ^∗∗∗∗^*p* < 0.0001. All determinations were made in duplicate.

HFD rats demonstrated significantly elevated IL-1*β* concentration and CAS-3 activity in the parotid (↑141%, *p* < 0.0001, and ↑82%, *p* = 0.0008, respectively) and submandibular glands (↑130%, *p* < 0.0001, and ↑68%, *p* = 0.0025, respectively) compared to the controls (Figure 5).

### 3.7. Oxidative and Nitrosative Stress in the Parotid and Submandibular Glands

**Figure 6:**
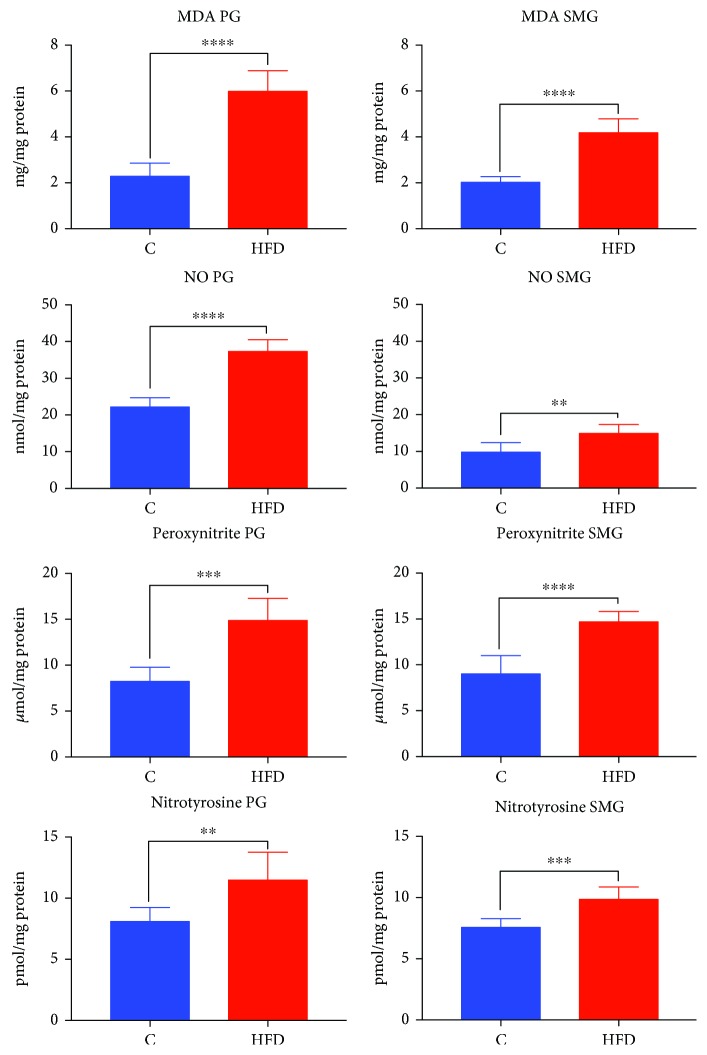
Oxidative and nitrosative stress biomarkers in the parotid and submandibular glands of HFD (*n* = 10) and control (*n* = 10) rats. PG: parotid glands; SMG: submandibular glands; C: control group; HFD: high-fat diet group; MDA: malonaldehyde; NO: nitric oxide. ^∗∗^*p* < 0.005, ^∗∗∗^*p* < 0.0005, and ^∗∗∗∗^*p* < 0.0001. All determinations were made in duplicate.

The parotid (↑158%, *p* < 0.0001) and submandibular (↑103%, *p* < 0.0001) glands of HFD rats showed significantly higher MDA concentration compared to those of the control group (Figure 6).

In the parotid and submandibular glands of HFD rats, the concentrations of NO (↑67%, *p* < 0.0001, and ↑52%, *p* = 0.0019, respectively), peroxynitrite (↑79%, *p* = 0.0001, and ↑61%, *p* < 0.0001, respectively), and nitrotyrosine (↑41%, *p* = 0.0062, and ↑29%, *p* = 0.0004, respectively) were significantly increased compared to those in the control group (Figure 6).

### 3.8. Submandibular vs. Parotid Glands

#### 3.8.1. Control Rats

XO and CAS-3 activities, GSH and NO concentration, and redox ratio were significantly higher in the parotid vs. submandibular glands (*p* < 0.0001, *p* < 0.02, *p* < 0.02, *p* < 0.0001, and *p* < 0.0001, respectively) (Table 2).

The control rats demonstrated significantly higher Complex I and GSSG concentrations in the submandibular glands compared to the parotid glands (*p* < 0.0005 and *p* < 0.0004, respectively) (Table 2).

#### 3.8.2. HFD Rats

The activities of NOX (*p* < 0.008), XO (*p* < 0.0001), CS (*p* < 0.004), and CAS-3 (*p* < 0.003), and DCFH-DA production (*p* < 0.0009), as well as concentrations of GSSG (*p* < 0.005), MDA (*p* < 0.004), NO (*p* < 0.0001), IL-1*β* (*p* < 0.008), and ADP/ATP ratio (*p* < 0.05), were significantly increased in the parotid vs. submandibular glands (Table 3).

H_2_O_2_ production (*p* < 0.0009), GSH concentration (*p* < 0.03), and redox ratio (*p* < 0.02) were higher in the submandibular compared to the parotid glands (Table 3).

#### 3.8.3. Correlation

A negative correlation was observed between mitochondrial H_2_O_2_ production and Complex I activity in the parotid (*p* = 0.01 and *r* = 0.67) and submandibular (*p* = 0.02 and *r* = 0.59) glands of HFD rats.

A positive correlation was noted between DCFH-DA concentration and NOX activity, while a negative relationship was observed between peroxynitrite concentration and Complex I activity in parotid glands of HFD rats.

## 4. Discussion

The previous published reports strongly suggest that OS in salivary glands is associated with IR [1, 3, 4]. However, the cause of increased production of free radicals and, consequently, the occurrence of oxidative stress in salivary glands in the course of IR are still unknown. In the scope of the experiment described above, we investigated the function of salivary gland mitochondria, mitochondrial and cytoplasmic ROS production, and other enzymatic endogenous sources of ROS, as well as selected markers of nitrosative and oxidative stress.

HFD is known to promote obesity and provides an excellent model for studies on diet-induced metabolic changes and oxidative stress in the course of IR [1, 3, 4, 36, 37].

The data presented herein indicate that after 8 weeks of feeding a high-fat diet, rats were overweight, hyperglycemic, and hyperlipidemic compared to the control group. They demonstrated hyperinsulinemia and significant increase in HOMA-IR, which is consistent with our previous reports and confirms the development of IR [4, 37]. These metabolic alterations were associated with systemic (data not shown) and salivary gland OS.

The paradox associated with the presence of oxygen in the atmosphere is that, on the one hand, oxygen metabolism provides aerobic organisms with more energy compared to anaerobic metabolism but, on the other hand, gradual reduction of oxygen entails the production of ROS. The major sites of ROS production in the body are mitochondria, where electrons are normally transferred through mitochondrial electron transport chain (ETC) in order to reduce molecular oxygen. The product of this reaction is water. During the above-mentioned transport, about 0.2–2% of electrons “escape” from ETC, which results in the production of a superoxide anion [38–40]. ROS are also produced in various cellular sources, including NOX, XO, cyclooxygenases, and lipoxygenases [41]. Among all the said sources, ETC and NOX, especially ETC, are considered the main sources of cellular ROS.

In our experiment, HFD diet was associated with increased mitochondrial ROS production in both types of glands of HFD rats. However, we observed a higher concentration of H_2_O_2_ in the mitochondria of the submandibular glands than in the parotid glands of rats fed a high-fat diet. The precise mechanism responsible for electron leakage is still unknown, but this phenomenon may be partially attributed to substrate excess, and in part also to ETC dysfunction.

Chronic exposure to HFD is connected with continued and increased delivery of free fatty acids to mitochondria. Increased flow of FFA to mitochondria increases oxygen consumption and production of oxide anion as a consequence of intensified reduction of the respiratory chain complexes [14].

The available data indicates that the potential sites of mitochondrial ROS formation are located in Complex I [42] and Complex II+III [43] and the most intensive production of hydrogen peroxide originates from electron flow from Complex I to Complex II [44]. Furthermore, it is known that reduced activity of ETC (electron transport chain) complexes increases mitochondrial ROS production [45, 46]. In our experiment, high-fat feeding resulted in greater inhibition of Complex I activity compared to Complex II+III in both glands of HFD rats. ROS accumulation in the mitochondria of both types of salivary glands in HFD rats was inversely related to Complex I activity, which may suggest that ROS are produced in the mitochondria mainly by defective Complex I, and—to a lesser extent—in Complex II+III. It has been proven that impairment of Complex I activity is a result of posttranslational modification and/or reduced expression [46]. The supposed reasons for this phenomenon include disturbed cAMP-dependent phosphorylation of the 18 kDa subunit of Complex I [46], reduced synthesis of its subunits, their defective assembly, and stable or increased oxidation and the resulting degradation of the protein core [45]. The reason for lower mitochondrial ROS concentration in the parotid vs. submandibular glands of HFD rats, with similar % inhibition of their mitochondrial complexes, is difficult to define. Evidence showed that the parotid not the submandibular glands are highly aerobic organs and have a highly efficient mitochondrial antioxidant system, comparable to that of liver mitochondria, capable of scavenging a large part of the produced ROS [47, 48].

The measurement of apoptosis confirmed the presence of salivary gland damage as demonstrated by increased caspase-3 activity in both glands of HFD rats. This observation may be the result of the inability of salivary gland cells to maintain appropriate ATP concentration as it was previously shown that a decreased ATP level leads to intensified hepatocyte apoptosis [49]. It should be stressed that electron transport and oxidative phosphorylation are closely interconnected. It has been demonstrated that impeded functioning of the respiratory chain results in weakened ATP production [18, 50]. Moreover, CS is a key enzyme associated with the functioning of the tricarboxylic acid cycle, and its reduced activity may reflect inhibited activation of this cycle and thus indicate further decrease in ATP production as well as reduced metabolic activity of the organ [13]. Indeed, our results showed increased ADP/ATP ratio in both glands of HFD rats. It has been evidenced that apoptotic death of acinar cells in salivary glands leads to acute impairment of their function [51], which—with weakened anabolic processes responsible for replacing damaged or lost cellular elements—could be the reason for the observed drop in the synthesis/secretion of protein (↓protein concentration in both types of salivary glands) and reduced stimulated salivary secretion. We also noticed increased COX activity in both types of salivary glands of HFD rats compared to those of the controls, which could be the result of ATP deficiency. Under physiological condition, ATP bounds to and thus inhibits COX with a subsequent low ROS formation [52]. On the other hand, it could be that cellular disturbances accompanying IR abolishes ATP-dependent inhibition of COX resulting in excessive ROS production.

Previously, we found profound salivary gland fat infiltration in that model [4]. Lipid droplets were more pronounced in the parotid glands [4] and were mainly attributable to increased lipolysis and impaired oxidation of the salivary gland lipid *β* [37]. It was shown that, with the consumption of high-fat diet, adipocytes boost the release of monocyte chemoattractant protein-1 (MCP-1). MCP-1 has a chemotactic effect on monocytes that flow to the adipose tissue and are transformed in tissue resident inflammatory M1 phenotype macrophage [53]. Macrophage infiltration further contributes to the formation of prooxidative environment by increasing NOX activity, which is a major source of ROS in adipocytes. In one study, NOX activity was demonstrated to be positively correlated with the amount of adipose tissue [54]. The treatment with NOX inhibitor reduced the lipid peroxidation level as well as H_2_O_2_ production in adipose tissue [55]. NOX is also activated by high concentrations of glucose [56] and free fatty acids [57], all of which were increased in the serum of HFD rats. Bearing in mind intensified adipose degeneration in the parotid glands and, to a smaller extent, in the submandibular glands, it is not surprising that the increase in NOX and DCFH-DA activity was greater in the parotid compared to the submandibular glands of HFD rats. The positive correlation between DCFH-DA and NOX activity in the parotid glands of HFD rats suggests that NOX is the main cytoplasmic source of free oxygen radicals. Nevertheless, other cytoplasmic sources of ROS, such as xanthine oxidase, cannot be excluded, particularly as we demonstrated a significant increase in xanthine oxidase content in both salivary gland types in HFD rats. Superoxide anions released by NOX are significant mediators in numerous oxidative chain reactions and can be converted into other free radicals. This may also trigger signal transduction pathways by nuclear factor *κ*B and increase the production of proinflammatory cytokines, which we observed as an increase in IL-1*β* concentration in both glands. Moreover, the superoxide anion can react with NO (also excreted by the stimulated macrophages) and transform into peroxynitrite. Indeed, in our experiment, high-fat diet caused increased peroxynitrite formation in both salivary glands of HFD rats. Moreover, the significantly decreased GSH level in both glands as well as the redox ratio (GSH^2^/GSSG) in the parotid glands of HFD rats indicates deficiency of the most important antioxidant molecule for the detoxification of peroxynitrite which acts as a potent biological oxidant [58]. Therefore, it is not surprising that considerable increase in 3-tyrosine nitration of salivary proteins was observed in both glands and was more pronounced in the parotid glands. Similarly, higher intensity of oxidative stress measured by the concentration of MDA was observed in the parotid glands of HFD rats, which is consistent with our earlier papers [1, 4].

The parameters of nitrosative stress were not determined in the mitochondrial fraction but in the gland homogenate; however, the observed negative correlation between peroxynitrite concentration and Complex I activity in the parotid glands of HFD rats may indicate 3-tyrosine nitration and inactivation of Complex I subunits and other mitochondrial proteins, also in the salivary glands. It had been previously proven that peroxynitrite can easily react with iron-sulfur clusters in enzyme complexes of the mitochondrial electron transport chain and that the liver tissue and mitochondrial proteins were highly nitrated in HFD-fed mice [45].

## 5. Conclusions

Eight-week intake of high-fat diet impairs mitochondrial function in both types of salivary glands by enhancing ROS production. ROS concentration was higher in the mitochondria of the submandibular glands.

The mitochondrial system as well as NOX participates in increasing oxidative and nitrosative stress in the both types of salivary glands. In our study, NOX activity, DCFH-DA production, protein nitration, and lipid peroxidation were more pronounced in the parotid glands of HFD rats.

## Figures and Tables

**Table 1:** General characteristics of rats.

	C	HFD	*p*
Final body weight (g)	279 ± 16.31	345.4 ± 15.7	**<0.0004**
BMI (g/cm^2)^	0.50 ± 0.1	0.67 ± 0.4	**<0.002**
Fasting glucose (mg/dL)	95.1 ± 8.9	169 ± 15.9	**<0.0001**
Fasting insulin (*μ*U/mL)	4.2 ± 0.1	58.6 ± 6.2	**<0.0001**
HOMA-IR index	1.4 ± 1.2	21.3 ± 1.6	**<0.0001**
Plasma FFA (*μ*mol/L)	65.5 ± 9.5	174.3 ± 10.2	**<0.0001**
Food consumption (g/day)	20.88 ± 0.8	15.39 ± 0.6	**<0.04**
Energy intake (kJ/day)	239.6 ± 3.2	338.9 ± 5.1	**<0.0006**
Parotid gland weight (mg)	88.1 ± 9.28	103.1 ± 7.61	**<0.03**
Submandibular gland weight (g)	194 ± 21.4	211.6 ± 20.18	ns
NWS (*μ*L/min)	0.40 ± 0.3	0.38 ± 0.7	ns
SWS (*μ*L/min)	118 ± 5.2	65.7 ± 9.2	**<0.0004**
Parotid gland total protein (*μ*g/mL)	3960 ± 293.6	3164 ± 167.5	**<0.0006**
Submandibular gland total protein (*μ*g/mL)	3693 ± 207	3124 ± 198	**<0.003**

C: control group; HFD: high-fat diet group; BMI: body mass index; HOMA-IR: homeostasis model assessment of insulin resistance; FFA: free fatty acids; NWS: nonstimulated salivary flow; SWS: stimulated salivary flow.

**Table 2:** Comparison between parotid and submandibular glands of the control rats (*n* = 10).

	C		
	PG	SMG	*p*
*ROS production*			
NOX (nmol/min/mg protein)	9.381 ± 0.74	9.851 ± 0.83	0.3734
XO (*μ*U/mg protein)	20.91 ± 2.43	8.451 ± 0.60	**<0.0001**
DCFH-DA (nmol/mg protein)	2.548 ± 0.29	2.681 ± 0.56	0.6814
*Mitochondrial activity*			
Complex I (mU/mg protein)	26.71 ± 1.48	34.02 ± 1.47	**0.0004**
Complex II (mU/mg protein)	24.22 ± 1.92	25.49 ± 0.55	0.2526
Complex II+III (mU/mg protein)	2.572 ± 0.20	2.593 ± 0.15	0.8755
COX (mU/mg protein)	21.35 ± 1.96	19.38 ± 1.52	0.1146
CS (mU/mg protein)	28.87 ± 1.03	30.87 ± 1.3	0.0518
H_2_O_2_ production (nmol/min/mg protein)	25.1 ± 3.23	23.7 ± 2.09	0.4404
ADP/ATP ratio	0.6038 ± 0.08	0.565 ± 0.07	0.3051
*Glutathione metabolism*			
GSH (nmol/mg protein)	1.1 ± 0.09	0.9409 ± 0.05	**0.012**
GSSG (nmol/mg protein)	0.06881 ± 0.001	0.09504 ± 0.001	**0.0003**
Redox rate	17.74 ± 2.67	37.26 ± 2.23	**<0.0001**
GPx (mU/mg protein)	4.648 ± 0.85	6.608 ± 0.47	**0.0003**
GR (mU/mg protein)	3.707 ± 0.76	3.017 ± 0.65	0.0951
*Oxidative and nitrosative stress*			
MDA (mg/mg protein)	2.34 ± 0.51	2.08 ± 0.19	0.3232
NO (nmol/mg protein)	22.54 ± 2.14	10.2 ± 2.19	**<0.0001**
Peroxynitrite (*μ*mol/mg protein)	8.387 ± 1.38	9.171 ± 1.83	0.4244
Nitrotyrosine (pmol/mg protein)	8.203 ± 1.04	7.675 ± 0.61	0.311
*Inflammation and apoptosis*			
IL-1*β* (pg/mg protein)	0.8482 ± 0.23	0.6239 ± 0.16	0.077
CAS-3 (*μ*mol/min/mg protein)	0.2814 ± 0.061	0.1979 ± 0.04	**0.0148**

PG: parotid glands; SMG: submandibular glands; C: control group; HFD: high-fat diet group; NOX: NADPH oxidase; XO: xanthine oxidase; DCFH-DA: 2,7-dichlorodihydrofluorescein diacetate assay for free radical production; COX: cytochrome c oxidase; CS: citrate synthase; H_2_O_2_: hydrogen peroxide; GPx: glutathione peroxidase; GR: glutathione reductase; GSH: reduced glutathione; GSSG: oxidized glutathione; MDA: malonaldehyde; NO: nitric oxide; IL-1*β*: interleukin-1*β*; CAS-3: caspase-3. All determinations were made in duplicate.

**Table 3:** Comparison between parotid and submandibular glands of the HFD rats (*n* = 10).

	HFD		
	PG	SMG	*p*
*ROS production*			
NOX (nmol/min/mg protein)	14.98 ± 1.57	12.41 ± 1.20	**0.0076**
XO (*μ*U/mg protein)	30.41 ± 1.55	12.88 ± 1.72	**<0.0001**
DCFH-DA (nmol/mg protein)	6.172 ± 1.04	4.168 ± 0.25	**0.0008**
*Mitochondrial activity*			
Complex I (mU/mg protein)	18.12 ± 1.77	18.03 ± 2.59	0.9502
Complex II (mU/mg protein)	26.61 ± 2.65	24.48 ± 2.98	0.2665
Complex II+III (mU/mg protein)	2.123 ± 0.16	2.05 ± 0.21	0.6038
COX (mU/mg protein)	30.38 ± 2.63	27.76 ± 1.41	0.0858
CS (mU/mg protein)	21.24 ± 1.54	18.79 ± 2.40	**0.0399**
H_2_O_2_ production (nmol/min/mg protein)	46.48 ± 5.75	62.48 ± 7.44	**0.0052**
ADP/ATP ratio	0.7425 ± 0.12	0.635 ± 0.07	**0.0448**
*Glutathione metabolism*			
GSH (nmol/mg protein)	0.5624 ± 0.25	0.7352 ± 0.16	**0.0234**
GSSG (nmol/mg protein)	0.1111 ± 0.001	0.08234 ± 0.01	**0.0042**
Redox rate	3.183 ± 1.99	27.31 ± 9.7	**0.0006**
GPx (mU/mg protein)	3.195 ± 0.62	6.185 ± 0.46	**<0.0001**
GR (mU/mg protein)	2.658 ± 0.74	3.508 ± 0.58	**0.0350**
*Oxidative and nitrosative stress*			
MDA (mg/mg protein)	6.04 ± 0.84	4.24 ± 0.54	**0.0039**
NO (nmol/mg protein)	37.67 ± 2.84	15.29 ± 2.02	**<0.0001**
Peroxynitrite (*μ*mol/mg protein)	15.03 ± 2.25	14.84 ± 0.97	0.8587
Nitrotyrosine (pmol/mg protein)	11.59 ± 2.16	9.967 ± 0.90	0.1209
*Inflammation and apoptosis*			
IL-1*β* (pg/mg protein)	2.056 ± 0.32	1.436 ± 0.20	**0.0071**
CAS-3 (*μ*mol/min/mg protein)	0.5071 ± 0.09	0.3216 ± 0.07	**0.0026**

PG: parotid glands; SMG: submandibular glands; C: control group; HFD: high-fat diet group; NOX: NADPH oxidase; XO: xanthine oxidase; DCFH-DA: 2,7-dichlorodihydrofluorescein diacetate assay for free radical production; COX: cytochrome c oxidase; CS: citrate synthase; H_2_O_2_: hydrogen peroxide; GPx: glutathione peroxidase; GR: glutathione reductase; GSH: reduced glutathione; GSSG: oxidized glutathione; MDA: malonaldehyde; NO: nitric oxide; IL-1*β*: interleukin-1*β*; CAS-3: caspase-3. All determinations were made in duplicate.

## Data Availability

The data used to support the findings of this study are available from the corresponding author upon request.
